# Five-Year Results of the Salto XT Revision Ankle Arthroplasty

**DOI:** 10.1177/10711007241264561

**Published:** 2024-07-29

**Authors:** Mads Sundet, Karen S. Gyllensten, Eva Dybvik, Kari H. Eikvar, Geir Hallan, Siri Lillegraven, Marianne Lund Eriksen

**Affiliations:** 1 Department of Orthopedic Surgery, Diakonhjemmet Hospital, Oslo, Norway; 2 Center for Treatment of Rheumatic and Musculoskeletal Diseases (REMEDY), Diakonhjemmet Hospital, Oslo, Norway; 3 The Norwegian Arthroplasty Register, Department of Orthopaedic Surgery, Haukeland University Hospital, Bergen, Norway; 4 The Norwegian Medial Association, Bergen, Norway; 5 Department of Clinical Medicine, University of Bergen, Norway

**Keywords:** total ankle replacement, revision arthroplasty, ankle fusion

## Abstract

**Background:**

The treatment of failed ankle replacements is debated, and little is published about the medium- and long-term results of revision implants. We wanted to examine prosthesis survival and physical function at least 5 years after insertion of the Salto XT revision prosthesis.

**Methods:**

All consecutive patients operated with a Salto XT revision prosthesis underwent clinical and radiologic examinations preoperatively and after 3, 12, 24, and 60 months. Complications and reoperations are described, and changes in patient-reported outcome measures and clinical scores are reported.

**Results:**

Thirty patients were operated with a Salto XT revision prosthesis between March 2014 and March 2017. Three of these were revised (1 to a fusion and 2 to a new prosthesis), and 3 patients were reoperated with screw removal. A concurrent subtalar fusion was performed on 13 patients, and there was 1 case of likely nonunion after these procedures, but no reoperations. The mean AOFAS score increased from 39.2 (95% CI 30.8-47.5) preoperatively to 75.1 (95% CI 67.3-82.9) after 5 years, and the mean improvement was 34.2 points (95% CI 23.8-44.6). Mean EQ-5D increased from 0.36 (95% CI 0.30-0.42) preoperatively to 0.74 (95% CI 0.64-0.85) after 5 years, an improvement of 0.34 (95% CI 0.19-0.49). Radiolucent lines were present in all but 3 patients. Five-year prosthesis survival was 93% (83.6-100).

**Conclusion:**

This is the first study to present medium-term results of this implant. We found good improvement in outcome scores and good implant survival, but also a high prevalence of radiolucent lines.

**Level of Evidence:** Level IV, case series.

## Introduction

Total ankle replacements (TARs) have a high rate of failure compared to replacements in knees and hips.^19^ Management of the failed TAR is controversial, and the main options are fusion or revision arthroplasty. Both options have a high rate of complications and unfavorable results for the patients. Fusions after failed TARs frequently fail to heal,^5,10^ and in a Swedish study only around half of the patients were satisfied with these procedures.^12^

Data on the results after revision TARs are limited. In a study from Swedankle, the Swedish joint replacement registry from 2015,^11^ revision TARs had a 5-year survival of 76% and a 10-year survival of 55%. The revision implants used in that study were not specified. Another registry study from the joint registry of England, Wales, and Northern Ireland showed a 3-year survival of revision arthroplasties of 87.7% and a 5-year survival of 77.5%.^9^ A recent review on the results after revisions of primary TARs found a reoperation rate of 26.9% in revisions to a new TARs, but they found very few studies on this subject.^8^

The Salto XT is a revision-specific implant,^16^ and the midterm performance of this implant has never been reported. The aim of this study is to present results at a minimum of 5 years of follow-up for the Salto XT revision ankle replacement.

## Methods

### Data Collection

Diakonhjemmet Hospital is the main center in Norway for ankle replacement surgery, both for primary cases and revisions. Data from all ankle replacements, both primary and revision, has been prospectively recorded in an internal ankle replacement database since 2013.

In the present study, all cases of revision TAR with any Salto XT component were included (30 patients). Patients were examined by physiotherapists with many years of experience in working with ankle arthroplasty patients preoperatively, and at 3 months, 1 year, 2 years, and 5 years postoperatively. The Norwegian Arthroplasty Register (NAR) was consulted for a quality control of the data, and they found the exact same patients registered from our hospital, and found no reoperations performed at other hospitals with this specific implant. Death dates were imported to the NAR from the Norwegian Population Register.

### The Salto XT Revision Arthroplasty

The Salto XT revision ankle prosthesis (Smith & Nephew, Watford, UK) is an implant specifically designed for revision of failed ankle replacements.^16^ It is a fixed-bearing implant based on and compatible with the Salto Talaris primary TAR (Smith & Nephew). Thus, it is possible to exchange one component of the TAR and leave the other if a Salto Talaris was the primary TAR. The metal components are made of cobalt-chromium and are coated with plasma-sprayed titanium, while the insert is made from ultrahigh-molecular-weight polyethylene. The tibial component has a 40-mm stem with the possibility of screw fixation, whereas the talar component has 2 types of stems: a 10.2-mm hollow stem for talar fixation, and a 55-mm stem penetrating the subtalar joint. There are 2 different versions of the latter stem, one with a 45-degree angulation from the talar cut and the other with a 35-degree angulation to allow for the talar component to replace bone defects in the posterior talus. The tibial component is interchangeable between the sides, whereas the talar component is specific for the right and left sides.

The procedures were performed by 2 foot and ankle surgeons (M.L.E. and K.E.) who had been operating TARs for more than 5 years before starting with this implant. The operation was done through the preexisting anterior approach, according to the general operative technique provided by the manufacturer, also described in a paper by Roukis.^16^ When the stemmed talar component was inserted and a subtalar fusion was not done previously, the subtalar fusion was performed through a separate sinus tarsi approach. All patients received antibiotic prophylaxis according to local procedures and received injections of low-molecular-weight heparin for at least 10 days to prevent venous thrombosis.

The patients were mobilized with crutches before discharge, approximately on the third postoperative day. The patients’ ankles were initially immobilized for 6 weeks—first 3 weeks in a cast, thereafter 3 weeks in a boot. During the first 3 weeks, the patients were allowed 10-15 kg of weightbearing; after that, they gradually increased the weightbearing until full weightbearing at 6 weeks postoperatively.

### Outcomes

The American Orthopaedic Foot & Ankle Society (AOFAS) score was the primary endpoint of the study, and this was routinely collected at every visit by an experienced physiotherapist. The minimal clinically important difference (MCID) for the AOFAS score is not established for ankle and hindfoot pathologies. For hallux valgus surgery, one publication calculated the MCID for the AOFAS score with several methods, and found results ranging from 7.9 to 30.2,^3^ and these upper and lower limits were both used in the analysis. The Manchester-Oxford Foot Questionnaire (MOXFQ) was introduced gradually to our registry during the follow-up and has mostly been recorded for the later visits. The EuroQoL-5D-3L (EQ5D) and a visual analog scale assessing health-related quality of life (VAS-QL) were completed at each visit, in addition to range of motion in the ankle. All the patients who were alive, not rerevised or lost to follow-up, had radiographs at 1 and 5 years, and these were reviewed by 2 experienced foot and ankle surgeons not involved in the clinical treatment of the patients (M.S. and K.G.), without reviewing the clinical scores or patient history. If there were discrepancies in the evaluations; the radiographs were reviewed and discussed, and an agreement was reached. The presence of periprosthetic lucencies or cysts were assessed; a radiolucent line was defined as a hypodense zone in the bone-implant interface, whereas a cyst was defined as a hypodense zone greater than 5 mm in diameter in proximity to the implant. CT scans had been performed in some patients on clinical indications, but not systematically. Complications and adverse events were investigated by reviewing the entire patient file until December 31, 2022.

### Statistical Analyses

Patients who were re-revised before 5 years did not participate in the 5-year data collection, but contributed data to other analyses. Demographic data and details about types of surgeries were summarized with percentages. Means were reported with 95% CIs, and *t* tests were performed to compare preoperative and postoperative scores. No imputation was done for missing values, and no sensitivity analyses were performed. *P* values less than .05 were considered significant. Implant survival was calculated using Kaplan-Meier analysis, and the patients were followed from their operation dates until death, revision, or to the end date December 31, 2022. The statistical analyses were performed with Stata BE 17.0 (StataCorp LLC, College Station, TX). The manuscript is prepared according to the STROBE guidelines.^20^

### Ethical Issues

The study is performed according to the standards of the Helsinki declaration. The data are collected from an internal quality database on ankle replacements, and according to Norwegian regulations on quality databases a written informed consent from the patients is not mandatory. The patients were informed about the data collection and the intended use of the data, and the database is approved by the hospital’s data protection officer and internal research board. The patients who have radiographs printed in the article has consented to this verbally.

## Results

### Demography and Implants

**Figure 1.**
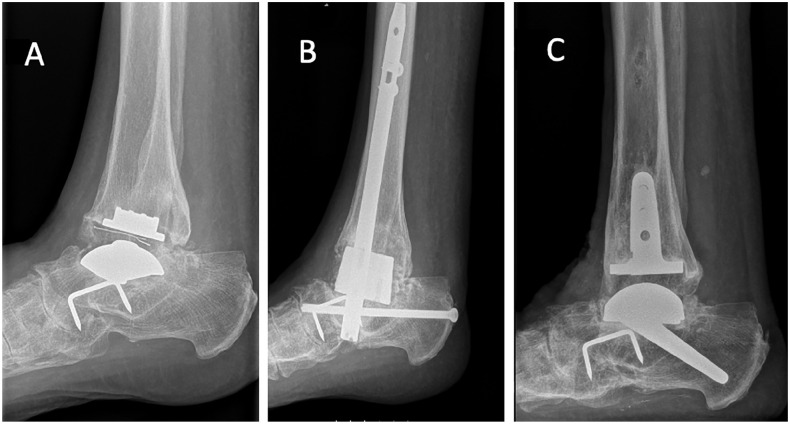
(A) Patient with failed STAR prosthesis that was fused with a retrograde nail in 2012. (B) This ended in a nonunion. (C) The prosthesis was revised to Salto XT in 2014, and in 2019 the patient was very satisfied with a MOXFQ of 15.6, AOFAS score was not recorded. AOFAS, American Orthopaedic Foot & Ankle Society ankle-hindfoot score; MOXFQ, Manchester-Oxford Foot Questionnaire.

Between March 2014 and March 2017, 30 patients were operated with the Salto XT revision ankle arthroplasty. All patients had failed previous TAR, but 1 of the patients had a takedown of a nonunited retrograde nailing after removal of a TAR (Figure 1). Fifteen of the patients (50%) were male, and the mean age was 60.2 years (SD 12.3). Table 1 shows information about previous implants and causes of revision.

**Table 1.** Characteristics of the Patients Included in the Study (N = 30).

Characteristics	Mean ± SD or n (%)
Age, y	
Male	57 ± 12.0
Female	64 ± 12.2
Body mass index	
Male	26.8 ± 2.4
Female	24.5 ± 5.1
Type of initial arthroplasty	
STAR	23 (76%)
Salto Talaris	4 (13%)
Mobility	1 (3%)
Rebalance	1 (3%)
CCI	1 (3%)
Cause of revision surgery	
Loosening	10 (33%)
Pain	8 (27%)
Instability/malalignment	4 (13%)
Wear	4 (13%)
Cysts	3 (10%)
Preoperative dorsiflexion, degrees	
Missing	10
0	2 (10%)
5	6 (30%)
10	10 (50%)
15	1 (5%)
20	1 (5%)
Dorsiflexion after 5 y, degrees	
Missing	9
0	3 (14%)
5	6 (28%)
10	7 (33%)
15	5 (24%)

Abbreviation: CCI, Charlson Comorbidity Index.

**Figure 2.**
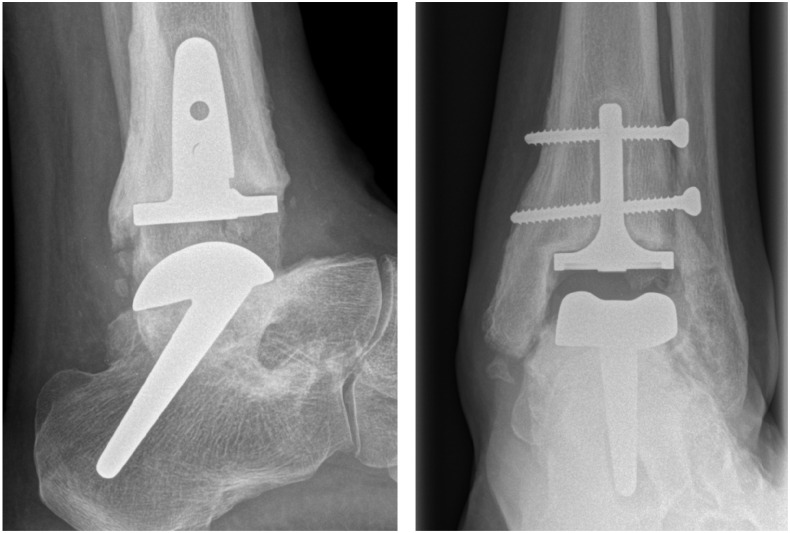
Forty-five-degree stem with healed subtalar fusion, breakage of screws and radiolucency around the tibial component. Good function after 5 years with an MOXFQ score of 17.2 and AOFAS score of 70. AOFAS, American Orthopaedic Foot & Ankle Society ankle-hindfoot score; MOXFQ, Manchester-Oxford Foot Questionnaire.

**Figure 3.**
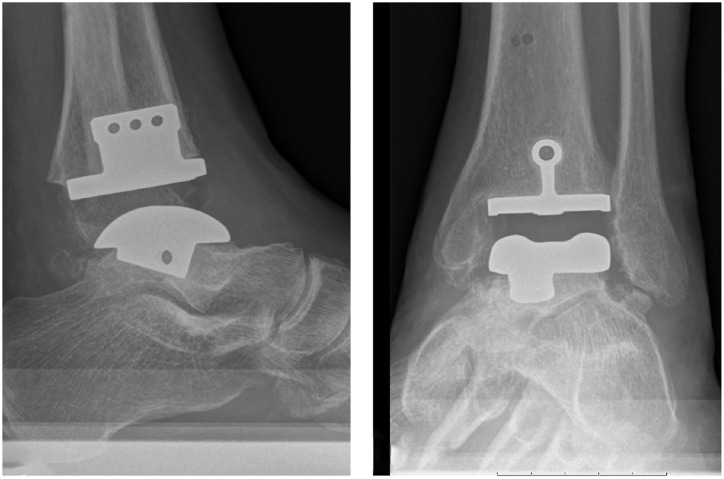
Short stem talar Salto XT with primary Salto Talaris tibia.

**Figure 4.**
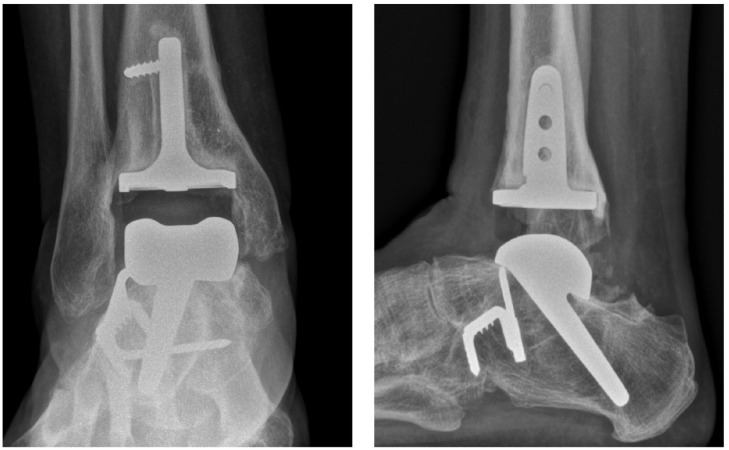
Thirty-five-degree talus stem through preexisting subtalar fusion, radiologic lucency, and screw breakage in tibia. Poor function after 5 years with a MOXFQ of 61 and an AOFAS score of 50. AOFAS, American Orthopaedic Foot & Ankle Society ankle-hindfoot score; MOXFQ, Manchester-Oxford Foot Questionnaire.

There were 15 long 45-degree stem talar components, 8 long 35-degree stem talar components, and 4 short-stem talar components, and 3 patients had a retained Salto Talaris talus component from the primary arthroplasty. In the tibia, 6 had a standard Salto Talaris, 4 of them retained from the primary arthroplasty, whereas the rest had a stemmed Salto XT. In 13 patients, a subtalar fusion was performed during the same procedure, and these fusions were stabilized with the long-stemmed implant alone and no additional screw fixation. Fourteen patients had screw fixation of the tibia. Figures 1 to 4 show different configurations of the implants.

### Reoperations and Complications

At 5 years, 4 patients had died of unrelated causes, 1 patient did not want to attend follow-up, and 2 had a clinical and radiologic follow-up without clinical scorings. One patient had done an ankle fusion (FAI reoperation type 12d) and 1 had done a revision of the tibia (FAI reoperation type 12a). Two other patients underwent a screw removal (FAI reoperation type 2); otherwise there were no other reoperations. One patient did a component revision (FAI reoperation type 12c) after 5.5 years, but this patient was not scored with MOXFQ or AOFAS after 5 years. Two patients died between the 5-year follow up and December 31, 2022, again of causes that were unrelated to the ankle surgery. There were no complications recorded except for the 3 cases of aseptic loosening, which is a high-grade complication according to the Glazebrook classification.^4^ Nine of the 14 patients with screw fixation of the tibia had screw breakage, but this is usually not classified as a complication. All revisions and reoperations are described in Table 2.

**Table 2.** Patients With Revisions of the Salto XT.

	Primary Diagnosis	Primary Implant	Reason for First Revision	Time to First Revision, months	Type of First Revision Arthroplasty	Type of Secondary Failure	Time to Second Revision/ Reoperation, months	Type of Second Revision/Reoperation
Patient 1	Posttraumatic	Salto Talaris	Loosening of tibia	18	Salto XT tibia, primary talar component retained	Loosening of tibia	8	Retrograde nailing
Patient 2	Posttraumatic	Salto Talaris	Loosening of tibia	12	Salto XT tibia, primary talar component retained	Pain. Possible loosening of tibia.	68	Revision arthroplasty (Invision/Inbone)
Patient 3	Hemochromatosis	CCI	Loosening of both tibia and talus	36	Salto XT tibia and talus, long stem	Loosening of tibia	32	1: Screw removal 2: New Salto XT tibia, with bone grafting
Patient 4								Screw removal
Patient 5								Screw removal

### Clinical Outcomes

**Figure 5.**
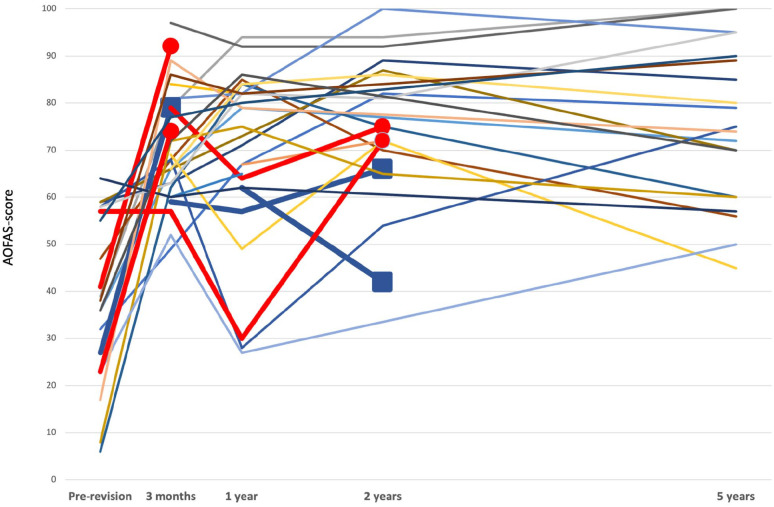
All registrations of AOFAS scores for individual patients, one line for each patient. Bold red lines represent 4 patients who died during the follow-up, whereas bold blue lines represent the 3 patients who were re-revised. Squares represent the last registration before revision and circles represent the last registration before death. [See online article for color figure.]

**Figure 6.**
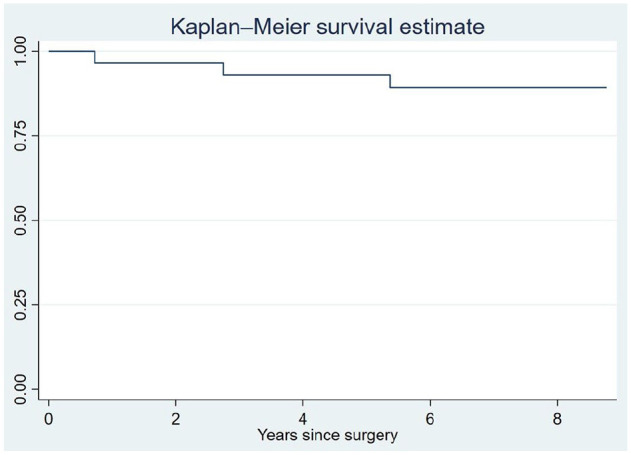
Kaplan-Meier curve for implant survival.

After 5 years, 21 nonrevised patients attended a physiotherapy follow-up. There were significant increases in all clinical scores, shown in Table 3. If the lower limit of the previously published MCID for AOFAS is used,^3^ 15 of the 16 patients who had both preoperative and 5-year AOFAS score available had an improvement of more than 7.9 points, whereas 1 patient had a worse AOFAS score after 5 years than preoperatively. If the upper limit of the MCID is used, 10 of the 16 patients improved more than 30.2 points. The complete AOFAS data are visualized in Figure 5, showing that there is a general increase in this score from preoperative, but that there is large variation in improvement between patients. The 5-year survival of the implant calculated by the Kaplan-Meier method was 93% (83.6-100) (Figure 6).

**Table 3.** Changes in Outcome Measures and Dorsiflexion.

Outcome	Preoperative Mean (95% CI)	12-mo Postoperative Mean (95% CI)	5-y Postoperative Mean (95% CI)	Change in Score From Preoperation to 5 y for Patients With Both Recordings (95% CI)	P Value
AOFAS	39.2 (30.8 to 47.5) (n = 20)	69.8 (62.2 to 77.4) (n = 26)	75.1 (67.3 to 82.9) (n = 20)	34.2 (23.8 to 44.6) (n = 16)	<.001
MOXFQ	58.6 (44.6 to 72.6) (n = 6)	42.5 (29.9 to 55.0) (n = 18)	25.0 (16.5 to 33.5) (n = 20)	−34 (3.4 to 65.9) (n = 5)	.04
EQ5D	0.36 (0.30 to 0.42) (n = 20)	0.69 (0.61 to 0.77) (n = 27)	0.74 (0.64 to 0.85) (n = 18)	0.34 (0.19 to 0.49) (n = 14)	<.001
VAS-QL	53.3 (45.1 to 61.8) (n = 20)	65.4 (57.6 to 73.2) (n = 26)	70.5 (61.5 to 79.5) (n = 19)	19.3 (4.7 to 33.9) (n = 14)	.013
Dorsiflexion	8.3 (6.1 to 10.4) (n = 20)	8.0 (6.6 to 9.4) (n = 27)	8.3 (6.0 to 10.6) (n = 21)	2.7 (–2.6 to 5.6) (n = 15)	.07

Abbreviations: AOFAS, American Orthopaedic Foot & Ankle Society ankle hindfoot score; EQ5D, EuroQoL-5D-3L; MOXFQ, Manchester-Oxford Foot Questionnaire; VAS-QL, visual analog scale assessing health-related quality of life.

### Radiologic Outcomes

After 1 year, 1 patient was dead and 29 patients were examined with radiographs. Twenty patients had radiolucent lines in the tibia (69%) and 13 patients in the talus (45%). After 5 years, the 23 remaining patients had radiographs, and 16 of these had radiolucencies in the tibia (70%) and 8 in the talus (35%). Only 3 patients had no radiolucent lines at any follow-up. There was no evidence in the data that suggested that the lucencies were symptomatic: At 5 years, 20 patients had recorded AOFAS scores, and 6 patients without lucencies had a mean AOFAS score of 66 (95% CI 45-86), whereas 14 patients with lucencies had an AOFAS score of 79 (95% CI 71-87) (P = .09). Of the 13 patients who underwent a concurrent subtalar fusion, 8 showed clear signs of healing on CT or radiographs at 5 years, whereas 4 were not conclusively healed and 1 had a possible nonunion, but had little symptoms from this. No patients developed cysts larger than 5 mm.

## Discussion

This article is the first to report medium-term outcomes of patients operated with the Salto XT revision arthroplasty, and one of few papers reporting clinical, radiologic, and patient-reported outcomes of revision TARs. We found a low revision and complication rate, and we found that the patients improved in MOXFQ, AOFAS, and EQ5D scores, whereas the mean range of motion was generally unchanged from preoperation. There were radiolucent lines around the implants at 1 or 5 years in all but 3 patients.

### Revision Rate

The 5-year survival of 93% was superior to the survival of the revision arthroplasties reported to Swedankle^11^ (76% at 5 years), which is not surprising because those patients were operated in an earlier time period, when revision-specific implants mostly were not available. Our results also compares favorably to the 5-year survival of primary TARs operated in Norway during the same period, as reported by the Norwegian Arthroplasty Register (85.2% [81.7%-88.7%]).^17^ The largest study that reports on the medium- to long-term results of a revision arthroplasty is a study on the Hintegra revision arthroplasty from 2013. In this study, 117 cases were reviewed and the 9-year survival rate was estimated to be 83%.^6^ Some shorter-term results has been published: Lachman et al.^14^ reported results from 29 revision arthroplasties with different types of implants. After a mean follow up-time of 3.3 years, 3 patients were revised. In recent years, the INBONE and Invision revision implants has been most extensively studied, and in a study from Munich, Germany, of 40 implants that had a mean follow-up time of 62 months, only 1 implant was revised.^15^ Another study from Exeter, United Kingdom, observed 23 INBONE and Invision implants and there were no revisions and very few complications after a minimum of 2 years.^7^ In the recent National Joint Registry study (England, Wales. and Northern Ireland), the Inbone implant had a 100% 3-year survival rate in 52 implants after TAR revision.^9^

### Radiolucent Lines

The study showed a large prevalence of radiolucent lines around both components of the Salto XT; only 3 patients had no such lines. There is reason to believe that component loosening may be present in some of these patients, and that this may be one of the reasons why the patient-reported outcome measure results in this cohort are so diverse. Interestingly, the presence of radiolucent lines was not associated with worse clinical scores. Also, some patients had lucency at 1 year but no lucency at 5 years. Despite the widespread presence of radiolucent lines, the revision rate was low. It is likely that the threshold for doing a new revision in the patient group is high, as re-revision surgery is complicated and entails a high risk of complications and/or a poor outcome. Also most of the patients have a long history of previous surgeries, and most of the patients had considerably better scores than in the pre-revision situation. The almost universal finding of radiolucent lines may be due to a suboptimal fixation of the implant to the bone, and polyethylene wear may also play a part. We have not been able to find accurate details about the manufacturing and sterilization procedures of the PE-implant. Both components are coated with plasma-sprayed titanium, and there was also the option of screw fixation of the tibia. Nine of the 14 patients with screw fixation had screw breakage, again suggesting a problem with bony ingrowth especially around the tibial component.

### Revision Arthroplasty vs Fusion

When a TAR fails, there are 2 surgical options: conversion to a fusion, or a revision arthroplasty. Both procedures are widely performed, but there is very little published evidence about the performance and outcomes of these procedures, especially when it comes to patient-reported outcome measures and clinical scores. Comparative studies are nonexisting. In Table 4, the largest available studies using MOXFQ or AOFAS as an outcome are summarized, allowing for some comparison to the present study. These data cannot be compared directly as there are large differences in follow-up time, number of patients lost to follow-up, and whether re-revised or nonunion patients are included in the follow-up. The superiority of any of these methods is not supported by available data today,^8^ and further studies are needed on this subject. Modern revision implants are also expensive, and there is a need for evaluating the cost-effectiveness of these methods.

**Table 4.** AOFAS and MOXFQ Scores After TAR Revisions From Other Studies (95% CI or SD, Otherwise Unreported in the Original Publication).

Type of Revision	Publication Year	Follow-up Time	MOXFQ Score	AOFAS Score
Fusion				
Sundet et al^18^: TTC fusions (n = 30)	2021	Mean 23 mo (range 6-46)	33.6	
Berkowitz et al^2^: TT fusions (n = 12)	2011	Mean 43.9 mo (range 8-106)		67 (SD 12)
Berkowitz et al^2^: TTC fusions (n = 15)^2^	2011	Mean 43.9 mo (range 8-106)		51.2 (SD 17)
Revision arthroplasty				
Lachman et al^14^: Revision arthroplasties (n = 29)	2019	Mean 3.3 y (range 2-8)		64.6
Hintermann et al^6^: Revision arthroplasties (n = 113)	2013	Mean 6.2 y (range 2-12)		72 (SD 19)
Jennison et al^8^: Revision arthroplasties (n = 23)	2022	2 y	29.1	
Behrens et al^1^: revision arthroplasties (n = 18)	2020	Mean 47.5 mo		Median 74.5 (range 26-100)
Present study: Revision arthroplasties (n = 30)		5 y	25.0 (16.5–33.5)	75.1 (67.3–82.9)

Abbreviations: AOFAS, American Orthopaedic Foot & Ankle Society ankle hindfoot score; MOXFQ, Manchester-Oxford Foot Questionnaire; TAR, total ankle replacement; TT, tibiotalar; TTC, tibiotalocalcaneal.

### Strengths and Limitations

One of the main strengths of the study is the standardized follow-up of the patients at fixed intervals. At 5 years, only 1 eligible patient was completely lost to follow-up, whereas 4 were dead and 3 were revised and not included in the 5-year results. Another strength is our independence from the designers and manufacturers of the implant under investigation. A limitation is that the follow-up was designed for clinical and not for scientific purposes, and the physiotherapists involved may have overlooked complications such as nerve or tendon injuries, and there are many missing registrations. The use of the AOFAS score as the primary outcome is not optimal, as it has been shown to have insufficient reliability and validity and its use is discouraged by the American Orthopaedic Foot & Ankle Society.^13^ Another limitation is the unsystematic use of CT scans, as for example subtalar nonunions may be overlooked with conventional radiographs. Also, the patients in this cohort have not been operated by a single, uniform “Salto XT” procedure. This is rather a family of different procedures under the same heading: some patients have stemmed implants, some have previous subtalar fusions, and some have retained a previous implant, and these subgroups are all too limited in size to draw conclusions from. Also, there is probably a survivorship bias in the clinical outcomes at 5 years because the revised patients were not included. Four patients were dead at this time, and this may also introduce bias to the results.

The generalizability of the results is also difficult to assess, also because the indication for a revision arthroplasty might vary from surgeon to surgeon.

## Conclusion

We have demonstrated a low revision rate and good improvements in physical function and health-related quality of life in patients operated with the Salto XT revision TAR system. Almost all patients had radiolucent lines around their implants, and bony ingrowth is possibly an issue with this implant. The study shows that in selected patients, revision TAR is a viable alternative to fusion for failed TARs. Very little has been published on this subject, and further research is necessary to clarify the optimal treatment strategy for these patients.

## Supplemental Material

sj-pdf-1-fai-10.1177_10711007241264561 – Supplemental material for Five-Year Results of the Salto XT Revision Ankle Arthroplasty Supplemental material, sj-pdf-1-fai-10.1177_10711007241264561 for Five-Year Results of the Salto XT Revision Ankle Arthroplasty by Mads Sundet, Karen S. Gyllensten, Eva Dybvik, Kari H. Eikvar, Geir Hallan, Siri Lillegraven and Marianne Lund Eriksen in Foot & Ankle International
